# Presynaptic Aβ40 prevents synapse addition in the adult *Drosophila* neuromuscular junction

**DOI:** 10.1371/journal.pone.0177541

**Published:** 2017-05-16

**Authors:** Begoña López-Arias, Enrique Turiégano, Ignacio Monedero, Inmaculada Canal, Laura Torroja

**Affiliations:** Department of Biology, Universidad Autónoma de Madrid, Madrid, Spain; University of Sydney, AUSTRALIA

## Abstract

Complexity in the processing of the Amyloid Precursor Protein, which generates a mixture of βamyloid peptides, lies beneath the difficulty in understanding the etiology of Alzheimer’s disease. Moreover, whether Aβ peptides have any physiological role in neurons is an unresolved question. By expressing single, defined Aβ peptides in *Drosophila*, specific effects can be discriminated *in vivo*. Here, we show that in the adult neuromuscular junction (NMJ), presynaptic expression of Aβ40 hinders the synaptic addition that normally occurs in adults, yielding NMJs with an invariable number of active zones at all ages tested. A similar trend is observed for Aβ42 at young ages, but net synaptic loss occurs at older ages in NMJs expressing this amyloid species. In contrast, Aβ42arc produces net synaptic loss at all ages tested, although age-dependent synaptic variations are maintained. Inhibition of the PI3K synaptogenic pathway may mediate some of these effects, because western analyses show that Aβ peptides block activation of this pathway, and Aβ species-specific synaptotoxic effects persists in NMJs overgrown by over-expression of PI3K. Finally, individual Aβ effects are also observed when toxicity is examined by quantifying neurodegeneration and survival. Our results suggest a physiological effect of Aβ40 in synaptic plasticity, and imply different toxic mechanisms for each peptide species.

## Introduction

Mounting evidences suggest that Alzheimer’s disease (AD) is primarily a disease of synaptic dysfunction [1], and place the amyloid Aβ peptide as the culprit of AD etiology. Indeed, *in vivo* and *in vitro* studies have shown that Aβ interferes with synaptic plasticity: it impairs excitatory transmission, inhibits LTP and enhances LTD [2,3]; it leads to spine retraction and synaptic loss [2,4,5]; and it causes robust behavioral deficits, particularly in learning and memory [2,6,7]. However, there are still important opened questions as to exactly which Aβ peptide forms trigger these synaptic alterations.

The latest prevalent version of the amyloid hypothesis states that AD arises from synaptic toxicity mediated by amyloid peptide (Aβ) soluble oligomers [8,9]. Proteolytic processing of the APP transmembrane protein by β and γ secretases generates a mixture of Aβ species that differ by few amino acids in their C-terminal sequence. Currently, it is unclear whether synaptotoxicity in AD is attributable to single specific Aβ species or rather to a quantitatively and/or qualitatively defined combination [10], or even if individual species harbor different toxic activities. *In vitro* experiments using biochemically defined Aβ species provide insights into these questions, but their relevance to the *in vivo* situation remains unresolved. Moreover, recent data suggest that Aβ may have a physiological role in synaptic plasticity: synaptic activity increases Aβ production [11], Aβ is necessary for memory formation [12], and low concentrations of Aβ enhance LTP and memory formation [12–15]. However, whether this is actually the case and its implication to AD is still undetermined [16].

*Drosophila* has proven a useful model to address these questions, because toxicity of specific amyloid species can be dissected *in vivo* [17,18]. Expression of amyloid peptides in *Drosophila* neurons recapitulates many of the pathological hallmarks of AD, including progressive neurodegeneration and behavioral deficits. In general, the severity of the behavioral phenotype correlated with peptide aggregation propensity and *in vivo* toxicity measured by longevity, neurodegeneration, and mobility assays [19,20–24]. However, Aβ40, which was proven nontoxic in these assays, induced early learning deficits in young flies [20,22].

The behavioral data suggest that all forms of Aβ, including Aβ40, produce synaptic defects early in life, yet electrophysiological and structural studies *Drosophila* have reported synaptic alterations only in response to Aβ42 or Aβ42arc [19,20,25,26]. However, the effects of Aβ40 on synaptic structure were assessed primarily in larval or pupal preparations. Because Aβ40 affects olfactory learning, but have no obvious effects on other behavioral processes such as climbing ability, it may exert subtle alterations in adult synaptic plasticity. Moreover, the progressive nature of AD cognitive decline indicates that Aβ synaptotoxicity is age-dependent. Therefore, we aimed at finding a model that could uncover small changes in age-related synaptic dynamics elicited by particular Aβ species.

The *Drosophila* adult NMJ should provide a simple, reproducible and quantifiable system to examine alterations on synaptic dynamics. A few studies in the adult NMJ have described age-dependent morphological changes [27,28]. These changes are consistent with an early period of synaptic refinement and maturation, analogous to that described in the fly antennal lobes [29] and mushroom bodies [30], and with a subsequent phase of age-related motor decline [31,32]. Thus, we hypothesized that the adult ventral NMJ would provide a model suitable for studying how particular Aβ peptides affect age-dependent synaptic remodeling.

In this study, we systematically compared the age-dependent synaptotoxic effect of presynaptic secretable forms of Aβ40, Aβ42, or Aβ42arc (a mutant form with increased aggregation propensity which causes early onset familial AD; [33]) in the *Drosophila* adult glutamatergic NMJ. We show that in wild type NMJs, there is an age-dependent variation in the number of active zones, which peaks at 15 days after eclosion. Our data shows that Aβ40 blocks these changes, so that it renders a slightly reduced, but constant number of synapses throughout adult life. On the contrary, both Aβ42 peptides cause net synaptic loss, albeit age-dependent variations are observed. These qualitative differences were apparent even in the presence of PI3K overexpression, which causes a dramatic increase in the number of synaptic contacts [34]. Our data provide new evidences of qualitative differences between the three Aβ peptides in their synaptotoxic activities, and suggest a physiological role for Aβ40 in synaptic plasticity.

## Materials and methods

### Fly culture

Fly stocks used were: *w*^*1118*^, *w*^*1118*^*;D42-GAL4* -drives expression in all motor neurons and in some neuronal populations in the adult brain [35]-, and *w*^*1118*^*;elav-GAL4* -drives panneural expression- (all from Bloomington *Drosophila* Stock Center http://flystocks.bio.indiana.edu/); *w*^*1118*^*;UAS-Aβ*_*42*_, *w*^*1118*^*;UAS-Aβ*_*42*_*Arc2E*, and *w*^*1118*^*;UAS-Aβ*_*40*_ (generously donated by Dr. Crowther D.C., [21]); *w*^*1118*^*;UAS-PI3K*^*92E*^ [36]. Lines harboring two *UAS* transgenes (Aβ and PI3K) were generated by standard genetic procedures.

For all experiments, males were analyzed from the progeny of crosses between *UAS* transgenic males and either *w*^*1118*^*;Gal4* (experimental flies) or *w*^*1118*^ (control flies) females. The Gal4 control males were collected from a cross between *w*^*1118*^*;Gal4* and *w*^*1118*^. All crosses were kept at 26°C and parents transferred to new tubes every 2–3 days to maintain similar crowding conditions. When adults started to eclose, tubes were emptied and males collected after 24h and kept in tubes at 26°C for aging.

### Longevity assay

Males of the specified genotypes were incubated at 26°C in groups of 20 in plastic vials and transferred every 2 days to fresh food. Death or lost flies were counted every 2 days. A minimum of five experiments per genotype was performed.

### Dissection and immunohistochemistry

**Fig 1:**
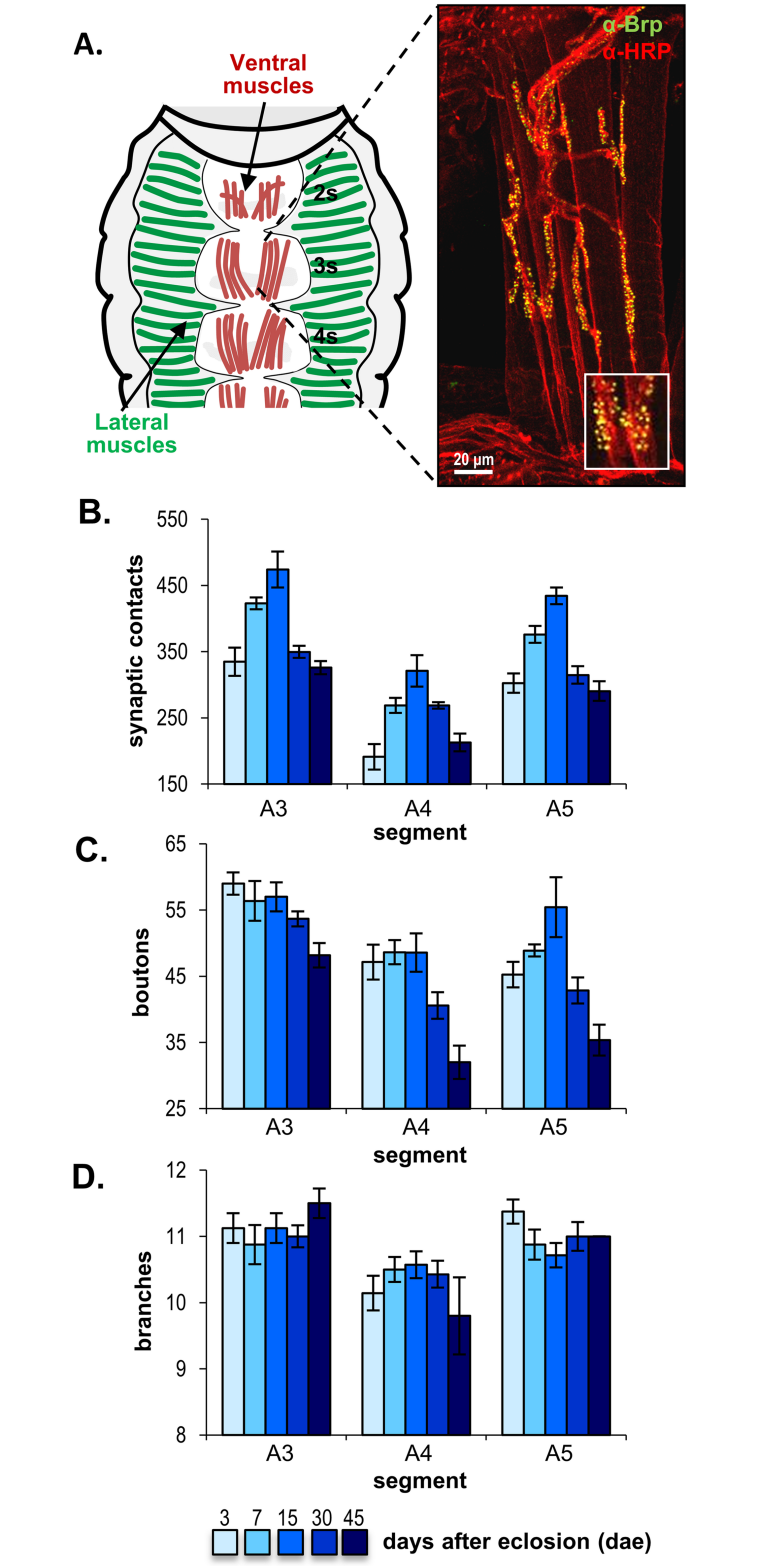
Age-dependent changes in the synaptic structure of the adult neuromuscular junction. **(A)** Schematic drawing of the anterior ventral abdomen (segments s2-s4), illustrating the position of the ventral abdominal muscles used for NMJ preparations. The accompanying image displays a representative confocal projection of a preparation labeled with anti-HRP (red) and anti-Brp (green). The inset shows active zones labeled with anti-Brp at a higher magnification. Scale bar = 20 μm. **(B-D)** Quantification of the number of active zones (synaptic contacts) **(B)**, boutons **(C)**, and branches **(D)** in control males in three different abdominal segments (A3, A4, and A5) at five ages (3, 7, 15, 30, and 45 days after eclosion (dae)). Statistical analyses showed that the three parameters showed segmental differences, but only synapses and boutons displayed age-dependent variations, that were similar in all segments. Number of synaptic contacts differed between the three segments, for boutons only segment A3 was statistically different from the other two segments, while branches was different in A4. Number of NMJs analyzed was between 7 and 9 for each segment/age pair, except for 45 days, which was 6 for A3, 5 for A4, and 3 for A5. Each data point represents the average ±SEM.

For neuromuscular junction (NMJ) analysis, ventral abdominal body-wall muscle preparations were dissected in Ca^2+^ free saline from 3, 7, 15, 20 and 30-day old adult males [37]. For assessing Aβ synaptotoxicity, we selected the ventral abdominal NMJ in the third abdominal hemisegment (Fig 1A). Samples were fixed in 4% formaldehyde in PBS, and immunostained with monoclonal antibody nc82 (anti-Bruchpilot 1:20, Developmental Studies Hybridoma Bank) visualized with α-mouse Alexa-488 (1:500, Invitrogen) and with Cy3-conjugated anti-HRP (1:200, Jackson Immuno Research). Anti-HRP signal reveals neuronal membranes, delimitating the motor neuron terminals. Bruchpilot is a CAST (CD3E-associated protein) homolog localized to the presynaptic specialization [38], and thus that was used to reveal active zones in the motor neuron terminal. Each Aβ-expressing genotype was processed simultaneously with its respective age-matched controls, to control for quantitative differences due to culture conditions.

### Clearing of brain tissue for neurodegeneration assays

15 and 20 day old adults were decapitated and fly brains dissected in PBS and fixed for 30 minutes in 10% formalin in phosphate buffer (Electron Microscopy Sciences). After 3 washes in PBS (5 minutes each), samples were permeabilized in cold methanol (Panreac) for 5 minutes, treated with clearing solution (1:2 Benzyl Alcohol:Benzyl Benzoate, Sigma Aldrich) for another 5 minutes as described [39], and mounted in the same solution in excavated slides.

### Image acquisition and quantification

Preparations were visualized using an Olympus IX70 confocal microscope. For body wall preparations, 1μm optical sections were taken. Stacks from hemisegments were used to quantify the number of Bruchpilot-containing active zones, with a macro-function developed in ImageJ that automatically selects the HRP-labeled motor terminal in each section and counts individual Bruchpilot-positive punctae while avoiding repeatedly counting the same synapse in contiguous sections. Number of boutons (ellipsoid enlargements of the HRP-labeled axon terminal) and branches (defined as number of HRP-labeled axonal branch tips per hemisegment) was manually quantified based on the anti-HRP signal using the ImageJ point picker tool.

Autofluorescence from whole brains was recorded with an argon 488 nm laser in 2μm optical sections. To quantify brain neurodegeneration, a macro function was developed in Fiji-win32 to automatically detect black circular areas (holes of neurodegenerated tissue) in each autofluorescent section of the brain and measure the area of neurodegeneration relative to the total brain area.

### Western blot

To measure total Aβ content, 10 heads of 15-day-old males of each genotype were homogenized in 20 μl of lysis/monomerization buffer (9M Urea, 1% SDS, 1mM EDTA, 25 mM Tris-HCL pH 7.5), sonicated with 3–4 pulses of 5 seconds, heated at 55°C during 1 hour, and centrifuged for 2 minutes. The supernatant was collected and proteins were separated by SDS–PAGE in 10–20% Tris–Tricine precast gels (Bio-Rad) and electroblotted onto 0.22 μm nitrocellulose membranes. The membrane was boiled in 1xPBS for 1 minute, and incubated with monoclonal anti-Aβ 6E10 (1:2000; Covance) and anti-αTubulin (1:2000, Sigma) and visualized by enhanced chemioluminescence (ECL, Amersham) in a Bio-Rad GelDoc XR+ system. Quantification was performed with ImageJ, and amount of Aβ signal relative to tubulin was averaged from three experiments.

Levels of phospho-Akt and phospho-GSK3β were used as readouts of PI3K activation. For this, 10 heads of 15-day-old males of each genotype were homogenized in 20 μl of RIPA buffer containing a cocktail of protease inhibitors and phosphatase inhibitors, and centrifuged for 2 minutes. The supernatant was collected and proteins were separated in Any kD TGX precast gels (Bio-Rad), and electroblotted onto 0.22 μm nitrocellulose membranes. Membranes were incubated with rabbit anti phospho(S505)-Akt or rabbit anti phospho(S9)-GSK3β (both 1:1000; Cell Signaling Technology) and visualized by enhanced chemioluminescence (ECL, Amersham) with a Bio-Rad Gel Doc documentation system. After stripping, the same membranes were used to measure rabbit anti *Drosophila* Akt or mouse anti GSK3β signals (both 1:1000; Cell Signaling Technology). Stripped membranes were finally probed with mouse anti Tubulin α (1:2000; Sigma-Aldrich). Quantification was performed with ImageJ, and the ratio p-S505Akt/Akt or p-S9GSK3β/GSK3β was averaged from four experiments.

### Data analysis

Parameters analyzed in NMJs were as follows: number of synaptic contacts, defined as Bruchpilot-positive active zones (also referred as synapses); number of boutons; number of branches; ratio synapses/bouton; and Aβ-induced synaptic reduction, which represents the percentage of active zones lost in each Aβ–expressing genotype with respect to the average number of synapses in age-matched controls ([“average #synaptic contacts in controls”–“#synaptic contacts in Aβ*”]/ “average #synaptic contacts in controls”). For genotypes expressing only Aβ peptides, Aβ-induced synaptic reduction was calculated relative to UAS controls, which showed a slightly lower synaptic count than Gal4 controls, and thus provided a conservative measure. For flies expressing Aβ and PI3K, synaptic reduction was calculated with respect to age-matched PI3K-expressing NMJs. To control for possible GAL4 titration effects that could reduce the levels of PI3K when expressed in conjunction with Aβ, nlsGFP instead of Aβ was co-expressed with PI3K (*w;UAS-PI3K/+; D42-Gal4 UAS-GFPnls/+*), and the number of synaptic contacts in 15 day-old flies was compared to age-matched *w;UAS-PI3K/+;D42-Gal4/+* flies. Statistical analysis showed no significant difference between both genotypes (number of synaptic contacts: 1035.7±47.3 for *w;UAS-PI3K/+; D42-Gal4/+* n = 10 NMJs (9 flies); 988.7±44.6 for *w;UAS-PI3K/+;D42-Gal4 UAS-GFPnls/+* n = 12 NMJs (7 flies); t_20_ = 0.72, p = 0.48,).

For NMJ parameters and neurodegeneration, we tested for the normality of all variables and their usual logarithmic transformation. Statistical significance was calculated for the normally distributed variables with a two-way ANOVA test followed by Bonferroni *post hoc*. Data that did not meet the normality assumption were analyzed with Kruskal-Wallis non-parametric test. Because there is not a widely accepted non-parametric test to analyze the effect of interaction between factors, we also performed ANOVAs on these data, considering that the results obtained with ANOVA and Kruskal-Wallis test were equivalent. The SPSS 15.0.1 software (SPSS Inc., USA) was used throughout. Data are presented as average ± SEM, and statistical functions are reported as F_g,N-1_ for the ANOVA analysis and H_g,N_ for the Kruskal-Wallis test, where N refers to the number of samples and g to the degrees of freedom. For regression analysis, we used the Real Statistics Resource Pack software (Release 3.5; Copyright (2013–2015) Charles Zaiontz; www.real-statistics.com) on Excel (Microsoft).

Differences in survival were analyzed using the Kaplan-Meier survival plots and log-rank analysis with the online survival analysis package OASIS [40]. Statistical significance was set at a corrected Bonferroni p value of 0.05.

## Results

### Age-dependent variations in the number of active zones in the adult Drosophila NMJ

Two studies in adult flies suggest that the fly NMJ undergoes morphological changes during the adult life [27,28]. While boutons enlarge throughout the adult life, branches elongate during the first two weeks but become shorter and thinner from this relatively young age onward [27]. The number of active zones was shown to increase during the first 5 days after eclosion [28], although this synaptic trait has not been analyzed at older ages. To characterize age-dependent synaptic structural changes in the *Drosophila* adult abdominal ventral NMJ (Fig 1A; [37]), we measured the number of branches, boutons, and Bruchpilot-positive active zones (referred to as number of synaptic contacts) in segments A3 to A5 at different ages between 5 and 45 days after eclosion (Fig 1), and analyzed the effect of segment, age, and their interaction on these variables with ANOVA (synapses and boutons) or Kruskal-Wallis (branches) tests.

The three parameters showed segmental differences (synapses: F_2,91_ = 82.735, p<0.001; boutons: F_2,91_ = 34.086, p<0.001; branches: H_(2,N = 106)_ = 17.807, p<0.001; Fig 1). However, only synapse and bouton number showed significant age-dependent differences (synapses: F_4,91_ = 36.178, p<0.001; boutons: F_4,91_ = 14.833, p<0.001; branches: H_(4,N = 106)_ = 1.343, p = 0.854;). Synapses continuously increased from 3 to 15 days after eclosion (dae), decreased from 15 to 30 dae and stabilized until at least 45-days of age (Fig 1B). For boutons, the number remained essentially constant until 15 dae, and decreased by 45 dae (Fig 1C). A similar temporal pattern was observed in all segments, as revealed by the lack of effect of the interaction segment*age in the statistical analyses (synapses: F_8,91_ = 1.237, p = 0.287; boutons: F_8,91_ = 1.450, p = 0.187; branches: F_8,91_ = 1.641, p = 0.124).

Our data shows that in the ventral abdominal NMJ, net synaptic addition occurs during the first two weeks after eclosion, while net synaptic elimination prevails afterwards in the aging fly. Thus, this adult NMJ arises as a simple suitable model for analyzing age-dependent alterations in synaptic dynamics due to Aβ expression. To differentiate physiological age-dependent changes from Aβ-induced synaptic alterations, the two temporal phases of synaptic remodeling will be referred to as “age-dependent synaptic addition” and “age-dependent synaptic elimination”.

### Presynaptic Aβ40 prevents synapse addition in the adult NMJ, while Aβ42 and Aβ42arc yield net synaptic elimination

**Fig 2:**
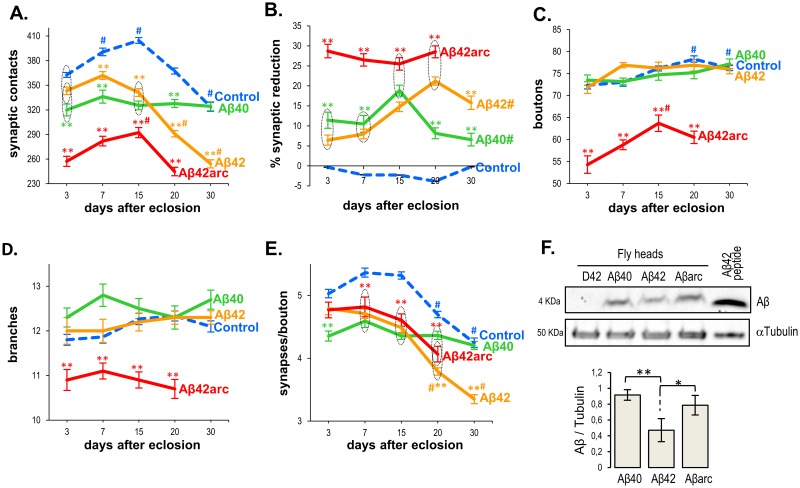
Age-dependent effects of three Aβ peptides on the synaptic structure of the adult neuromuscular junction. Parameters were quantified in the adult A3 ventral NMJ at 3, 7, 15, 20, and 30 days after eclosion (dae), in males expressing Aβ40, Aβ42, or Aβ42arc in motoneurons (w; D42-Gal4/UAS-Aβ*) or in control males (w; D42-Gal4/+ and w; UAS-Aβ*/+). For simplicity, only the Gal4 control, common to all experimental genotypes, is shown. Parameters measured were **(A)** number of synaptic contacts, **(B)** percentage of synaptic reduction measured as: (average number of synaptic contacts in control − number of synaptic contacts in Aβ*)/average number of synaptic contacts in control, **(C)** number of boutons, **(D)** number of branches, and **(E)** ratio of synaptic contacts per bouton. Each data point represents the average of 10 hemisegments of the same genotype/age ±SEM. Asterisks denote significant differences (** p<0.001) with their respective age-matched controls (Gal4 and UAS) and the other Aβ-expressing genotypes. Circled discontinuous lines surround data points that are not statistically different between each other but are statistically different with the other data points outside the circle. The symbol # indicates that *post hoc* analyses detected statistical differences (p<0.01) within each particular genotype between data from that age and data from 3 dae. Notice that the number of synapses in Aβ40-expressing NMJs (A) remains constant throughout all ages tested, while it shows age-dependent variations for the other genotypes. Number of flies analyzed, N, is larger than 20 for all ages in the control genotype except for 30 dae (N = 16). For all Aβ*-expressing genotypes N = 8, except for: *w; D42-Gal4/UAS-Aβ40* 20 dae (N = 9); *w; D42-Gal4/UAS-Aβ42* at 7 and 30 dae (N = 9) and at 15 dae (N = 7); *w; D42-Gal4/UAS-Aβarc* at 7 and 15 dae (N = 9) and at 3 and 30 dae (N = 7). **(F)** Detection of total Aβ peptide (4 KDa) by western blot in head homogenates from 15 day-old flies expressing Aβ40, Aβ42, Aβ42arc (Aβarc), or from Gal4 control flies (D42). The right lane contains purified Aβ42 mixed with extract from control fly heads. αTubulin (50 KDa) was used to control for total protein content. Amount of Aβ was normalized with respect to αTubulin. Each data point in the graph represents the average from 3 experiments ±SEM. ** p<0.01, * p<0.05.

**Fig 3:**
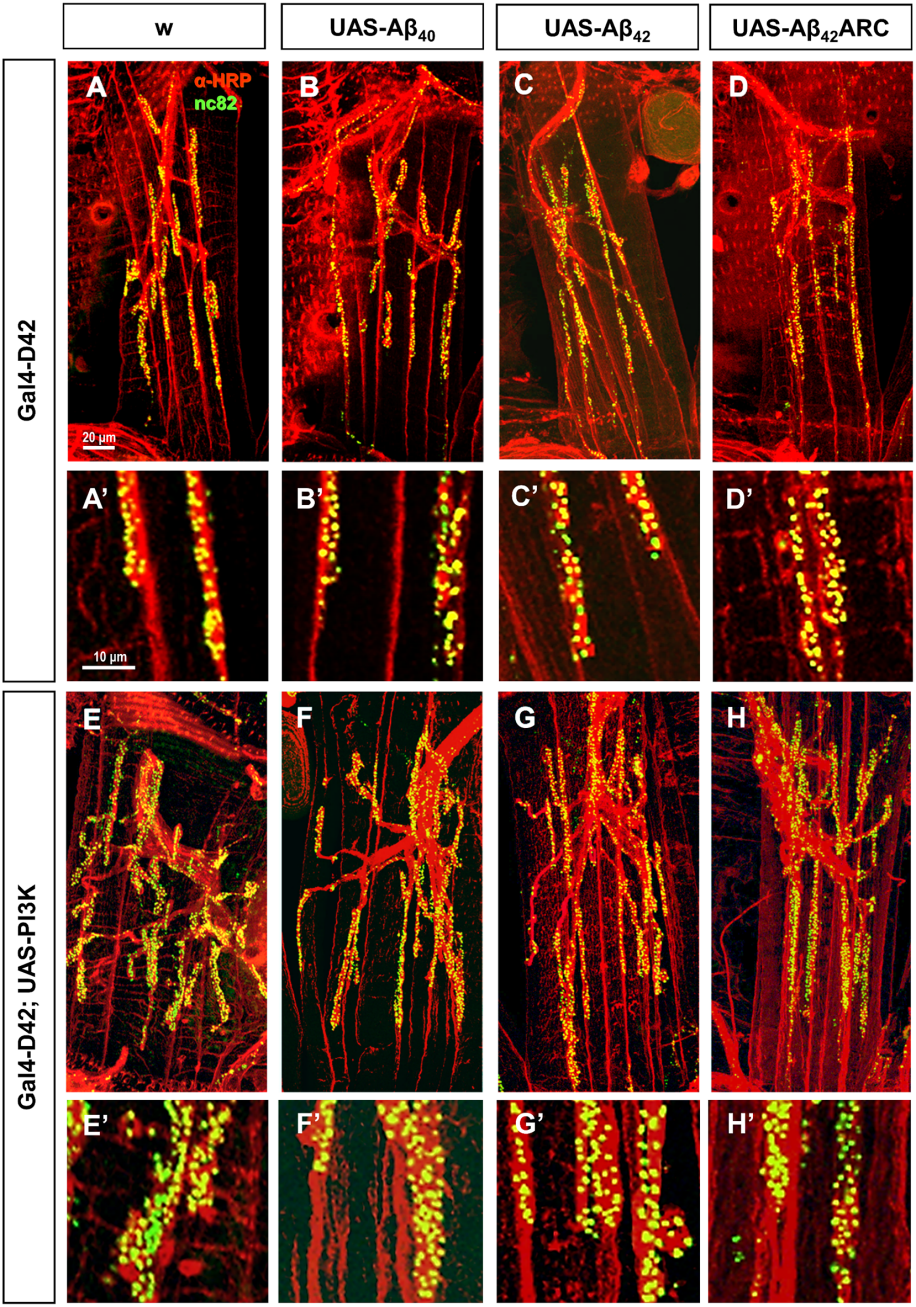
Morphology of 15-day-old fly NMJs expressing amyloid peptides, with or without elevated PI3K levels. Representative images showing active zones (green; anti-Bruchpilot) and neuronal membrane (red; anti-HRP) of A3 abdominal ventral NMJs from 15-day-old control males (w, A and E), and males expressing Aβ40 (B and F), Aβ42 (C and G), or Aβ42arc (D and H), with normal (A-D) or elevated (E-H) levels of PI3K. Genotypes analyzed were (A) *w; D42-Gal4/+*, (B-D) w; D42-Gal4/UAS-Aβ*, (E) *w; D42-Gal4/UAS-PI3K*, and (F-H) *w; D42-Gal4/UAS-PI3K*, UAS-Aβ*. The complete NMJ is shown in images A-H (scale bar 20 μm), and a higher magnification of part of the NMJ is shown in images A’-H’ (scale bar 10 μm).

To examine how Aβ influences synapses during ageing, we performed a systematic quantitative analysis comparing the effect of presynaptic Aβ40, Aβ42 and Aβ42arc on the morphology of the adult NMJ at different ages between 3 and 30 dae. Measures were taken at segment A3 and the effects of peptide (genotype), age, and their interaction, were statistically analyzed (Figs 2 and 3, Table 1).

**Table 1:** Summary of statistical results for the effect of different Aβ species on parameters of the adult neuromuscular junction.

	GENOTYPE		AGE		GENOTYPE*AGE	
SYNAPTIC CONTACTS	F_6,387_ = 243.612	p<0.001	F_4,387_ = 136.688	p<0.001	F_22,387_ = 9.189	p<0.001
	H_(6,N = 420)_ = 193.776	p<0.001	H_(4,N = 420)_ = 102.776	p<0.001		
BOUTONS	F_6,387_ = 92.355	p<0.001	F_4,387_ = 19.012	p<0.001	F_22,387_ = 1.513	p = 0.066
	H_(6,N = 420)_ = 108.558	p<0.001	H_(4,N = 420)_ = 64.466	p<0.001		
SYNAPSES/BOUTON	F_6,387_ = 51.033	p<0.001	F_4,387_ = 104.446	p<0.001	F_22,387_ = 4.849	p<0.001
BRANCHES	F_6,387_ = 26.225	p<0.001	F_4,387_ = 3.038	p = 0.017	F_22,387_ = 0.993	p = 0.472
	H_(6,N = 420)_ = 105.150	p<0.001	H_(4,N = 420)_ = 20.452	p<0.001		
SYNAPTIC REDUCTION	F_6,387_ = 244.835	p<0.001	F_4,126_ = 2.273	P = 0.061	F_7,126_ = 5.023	p<0.001
	H_(6,N = 420)_ = 254.330	p<0.001	H_(4,N = 420)_ = 0.818	P = 0.936		

Data on synaptic parameters measured in the adult ventral abdominal NMJs at segment A3, from seven genotypes (*w;D42-Gal4/+*, w;UAS-Aβ*/+, and w;D42-Gal4/UAS-Aβ*, being Aβ* either Aβ40, Aβ42, or Aβ42arc) and 5 ages (3, 7, 15, 20, and 30 days after eclosion (dae)) were used for the analysis. Data with normal distribution were analyzed with a two-way ANOVA (F) that tested the effect of two variables (age and genotype) and their interaction (age*genotype). For data with non-normal distribution, the effect of either genotype or age was analyzed with the non-parametric one-way Kruskal-Wallis test (H). In these cases, to analyze the effect of age*genotype interaction, a two-way ANOVA was also performed that tested the effect of genotype, age, and their interaction. Therefore, for non-normally distributed data, both ANOVA (F) and Kruskal-Wallis (H) tests results are included. A minimum of 10 NMJs was measured for each genotype and age (for details on number of flies analyzed, see Fig 2). The two subscripted numbers after F functions indicate the degrees of freedom for the between-group and within-group variance. For H function, the first subscript indicates the degrees of freedom.

Our results provide the first demonstration for a synaptic structural defect induced by Aβ40 on *Drosophila* synapses (Fig 2A; Table 1). All three peptides caused a significant synaptic reduction from 7 dae on. At 3 dae, Aβ40 and Aβ42arc also decreased synaptic contacts, while the number of active zones in Aβ42-expressing NMJs was intermediate between controls and NMJs with Aβ40, although it was not significantly different from either one. Consistent with its higher toxicity, Aβ42arc showed the strongest synaptic reduction (Fig 2A and 2B; Table 1) at all ages tested (at 30 days after eclosion, most Aβ42arc flies had died, and therefore this age was not analyzed). In contrast, Aβ42 induced a larger synaptic reduction than Aβ40 at 20 and 30 dae, but a similar effect at younger ages (Fig 2A and 2B). These peculiarities in Aβ-induced synaptotoxicity could not be solely attributed to quantitative differences in peptide levels, because western analysis showed similar levels of total Aβ40 and Aβ42arc at 15 dae (Fig 2F), yet a stronger synaptic reduction induced by the mutant peptide at this age. Overall, these data suggests that all amyloid species show an early tendency to decrease synapses, which is consistent with reported learning defects induced by the three Aβ peptides at young ages [22,23].

An intriguing observation was that the number of active zones in Aβ40-expressing NMJs remained constant throughout the ages tested. Indeed, statistical analyses showed an effect of the interaction between peptide type (genotype) and age on synapses (Table 1), denoting that Aβ expression alters the age-dependent changes of synapse number that occurs in physiological conditions. To further explore this question, we approximated the rate of synaptic change by the slope of the linear regression for the dataset covering the phase of age-dependent synapse addition (from 3 to 15 dae), and of age-dependent synapse elimination (from 15 to 30 dae; 15–20 dae for Aβ42arc)(Table 2).

**Table 2:** Linear regressions examining the rate of synaptic change in control and Aβ-expressing NMJs during two age intervals.

Age interval	Regression Analysis						Slope comparisons		
	Genotype	b (slope)	t	df	p	R^2^	t	df	p
3–15 dae	Control	2.825	6.778	98	<0.001*	0.319			
	Aβ40	0.202	0.245	28	0.806	0.002	2.921	126	0.004^#^
	Aβ42	-0.521	-0.825	28	0.417	0.024	3.925	126	<0.001^#^
	Aβ42arc	2.693	3.716	28	<0.001*	0.330	0.151	126	0.880
15–30 dae	Control	-5.135	-13.806	98	<0.001*	0.660			
	Aβ40	-0.111	-0.233	28	0.817	0.002	7.450	126	<0.001^#^
	Aβ42	-5.49	-10.712	28	<0.001*	0.804	0.520	126	0.604
	Aβ42arc (15–20 dae)	-9.5	-5.972	18	<0.001*	0.665	1.345	96	0.182

Linear regression analyses were performed for each genotype with data of the number of active zones pooled from 3 to 15 or from 15 to 30 (15 to 20 for Aβ42arc) days after eclosion (dae). We additionally performed six analyses comparing the slopes of the regression lines for each Aβ-expressing genotype with the slope obtained for the corresponding control genotype (“slope comparisons”).

* indicates that the slope of the regression line is significantly different from 0.

^#^ indicates that significant differences were found between the slopes.

In control NMJs, the age-dependent synaptic addition occurring from 3 to 15 dae rendered a slope with a positive value, while the age-dependent synaptic elimination that occurs between 15 and 30 dae yielded a negative slope (Table 2). The synaptic temporal pattern of Aβ40-expressing NMJs produced a slope which was not different from 0 for both age segments, and which was statistically different from both control regression lines (Table 2). Moreover, *post hoc* analyses identified Aβ40 as the only genotype in which the number of active zones did not differ between ages, while all other genotypes showed age dependent variations (Fig 2A). These data demonstrate that the effect of Aβ40 is qualitatively different from the other Aβ peptides. But more importantly, the results suggest that Aβ40 restrains the addition of new synapses that normally occurs during the first days of adult life.

For Aβ42-expressing NMJs, regression analysis revealed a modification in the temporal biphasic pattern of active sites. At young ages, from 3 to 15 dae, the rate of synaptic change was not different from 0 or from Aβ40, but differed from controls (Table 2). This suggests that in early adulthood, Aβ42 acts similarly to Aβ40, hindering synapse addition. In contrast, from 15 to 30 dae, age-dependent synaptic elimination occurred in Aβ42-expressing NMJs at a rate not different from controls (Table 2), suggesting a transformation in the synaptic action of this peptide with aging. Alternatively, the lower peptide content detected in Aβ42-expressing fly heads could account for its reduced early synaptotoxicity, while its age-dependent accumulation [21,22,24] would strengthen its toxic effects at older ages. In contrast to Aβ40 and Aβ42, expression of Aβ42arc yielded NMJs with an age-dependent rate of synaptic change statistically not different from controls at both age intervals (Table 2). These data demonstrate remarkable specific differences in the influence of age on the synaptic effects of each amyloid species.

For boutons and branches, only the Aβ42arc peptide showed a consistent significant effect (Fig 2C and 2D; Table 1). The reduction in the number of boutons (and/or branches) induced by Aβ42arc could account for its synaptic reduction, in which case the ratio of synapses per bouton should not differ from control NMJs. However, this ratio exhibited statistical differences between all Aβ-expressing genotypes, including Aβ42arc, and age-matched controls at most ages tested (Fig 2E), suggesting a specific impact of the peptide on synaptic contacts, independent of its influence on bouton and branch number.

### Aβ peptides induce synaptic reduction in adult NMJs enlarged by PI3K over expression

**Fig 4:**
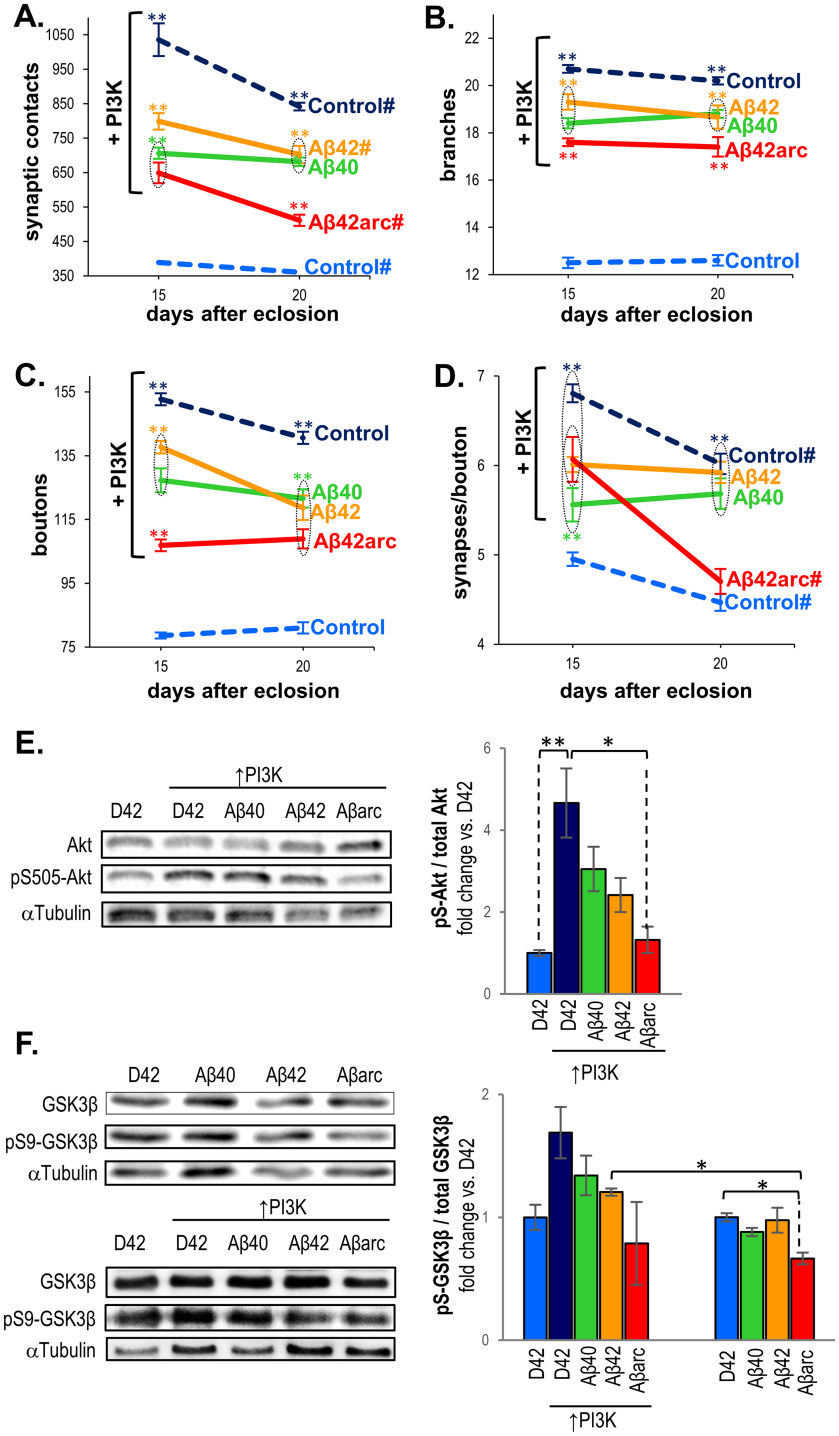
Specific effects of the three amyloid peptides on synaptic structure persists in expanded adult neuromuscular junctions over expressing PI3K. **(A-D)** Parameters were quantified in the adult A3 ventral NMJ at 15 and 20 days after eclosion, in males overexpressing in motor neurons: PI3K (*w;D42-Gal4/UAS-PI3K*), PI3K and either Aβ40, Aβ42, or Aβ42arc (w; D42-Gal4 / UAS-PI3K UAS-Aβ*), and in control males (w; D42-Gal4/+ and w; UAS-PI3K UAS-Aβ*/+). For simplicity, only the Gal4 control, common to all experimental genotypes, is shown (light blue line). Parameters measured were **(A)** number of synaptic contacts, **(B)** number of boutons, **(C)** number of branches, and **(D)** ratio of synaptic contacts per bouton. Each data point represents the average of 8–10 hemisegments of the same genotype/age ±SEM. Asterisks denote significant differences (** p<0.01) with their respective age-matched controls (Gal4 and UAS), and the other Aβ-expressing. The symbol # associated to the genotype label indicates that *post hoc* analyses detected statistical differences (p<0.01) between the two ages within that particular genotype. Notice that the number of synapses in Aβ40-expressing NMJs (A) remains constant from 15 to 20 dae, while it shows age-dependent variations for the other genotypes. Discontinuous circles surround data points that are not statistically different between each other but statistically different with the other data points outside the circle. The number of flies analyzed was N = 8, except for: w; *D42-Gal4/+* 15 dae (N = 9); *w; D42-Gal4 / UAS-PI3K* 15 dae (N = 9); *w; D42-Gal4 / UAS-PI3K UAS-Aβ40* 15 dae (N = 9); *w; D42-Gal4 / UAS-PI3K UAS-Aβ42* 20 dae (N = 7); *w; D42-Gal4 / UAS-PI3K UAS-Aβarc* 15dae (N = 9) and 20 dae (N = 7). **(E)** Representative blots showing detection of of pS505-Akt and total Akt in head homogenates from 15 day-old flies expressing PI3K (*w;D42-Gal4/UAS-PI3K*), PI3K and either Aβ40, Aβ42, or Aβ42arc (w; D42-Gal4 / UAS-PI3K UAS-Aβ*), and in control males (D42, *w; D42-Gal4/+*). Graph depicts fold changes of the ratio pS505-Akt/total Akt relative to control males. **(F)** Representative blots showing detection of pS9-GSK3β and total GSK3β in head homogenates from 15 day-old flies expressing each Aβ peptide (w; D42-Gal4 / UAS-Aβ*), PI3K and either Aβ40, Aβ42, or Aβ42arc (w; D42-Gal4 / UAS-PI3K UAS-Aβ*), PI3K in the absence of Aβ (*w;D42-Gal4/UAS-PI3K*), and in control males (D42, *w; D42-Gal4/+*). Graphs depict fold changes of the ratio pS9-GSK3β/GSK3β relative to control males. Anti Tubulin α was used for signal normalization in all blots. Each data point in the graphs represents the average from 4 experiments ±SEM. ** p<0.001, * p<0.01.

Presynaptic activation of the PI3K pathway promotes synaptogenesis both in *Drosophila* and mammals [34,41], while reducing the activity of the pathway, or increasing GSK3, a target inhibited by the pathway, has the opposite effect [34]. This pathway is altered in patients with Alzheimer’s disease, which show increased GSK3 activity, a condition that is thought to directly contribute to AD synaptic dysfunction [42,43]. Thus, we wondered whether PI3K activation might counteract Aβ induced synaptotoxicity. To test this, we overexpressed the PI3K catalytic subunit, Dp110, in motor neurons together with single Aβ species, and analyzed their combined effect on the adult NMJ. Synaptic morphological parameters were measured at 15 and 20 dae, two ages at which each Aβ peptide shows clear differences with respect to controls and to the other amyloid species (Figs 3 and 4, Table 3).

**Table 3:** Summary of statistical results for the effect of different Aβ species with normal or elevated PI3K levels, on parameters of the adult neuromuscular junction, at 15 and 20 days after eclosion.

	GENOTYPE		AGE		GENOTYPE*AGE	
SYNAPTIC CONTACTS	F_14,326_ = 457.206	p<0.001	F1,326 = 138.497	p<0.001	F14,326 = 8.309	p<0.001
	H(14,N = 356) = 268.191	p<0.001	H(1,N = 356) = 33.947	p<0.001		
BOUTONS	F_14,326_ = 292.856	p<0.001	F1,326 = 7.031	p = 0.008	F_14,326_ = 4.609	p<0.001
	H(14,N = 356) = 140.761	p<0.001	H(1,N = 356) = 1.006	p = 0.316		
SYNAPSES/BOUTON	F_14,326_ = 47.843	p<0.001	F1,326 = 110.890	p<0.001	F_14,326_ = 4.603	p<0.001
	H(14,N = 356) = 178.545	p<0.001	H(1,N = 356) = 58.535	p<0.001		
BRANCHES	F_14,326_ = 411.999	p<0.001	F1,326 = 1.233	p = 0.268	F_14,326_ = 1.014	p = 0.439
	H(14,N = 356) = 146.587	p<0.001	H(1,N = 356) = 0.017	p = 0.897		
SYNAPTIC REDUCTION	F_14, 326_ = 47.782	p<0.001	F_1,326_ = 6.649	p = 0.010	F_14,326_ = 9.270	p<0.0017

Data on synaptic parameters measured in the adult ventral abdominal NMJs at segment A3, from fifteen genotypes (*w;D42-Gal4/+*, w;UAS-Aβ*/+, *w;UAS-PI3K/+*, w;UAS-Aβ* UAS-PI3K/+, *w;D42-Gal4/UAS-PI3K*, w;D42/UAS-Aβ*, and w;D42-Gal4/UAS-Aβ* UAS-PI3K, being Aβ* either Aβ40, Aβ42, or Aβ42arc) and 2 ages (15 and 20 days after eclosion (dae)) were used for the analysis. Data with normal distribution were analyzed with a two-way ANOVA (F) that tested the effect of two variables (age and genotype) and their interaction (genotype*age). For data with non-normal distribution, the effect of either genotype or age was analyzed with the non-parametric one-way Kruskal-Wallis test (H). In these cases, to analyze the effect of age*genotype interaction, a two-way ANOVA was also performed that tested the effect of genotype, age, and their interaction. Therefore, for non-normally distributed data, both ANOVA (F) and Kruskal-Wallis (H) tests results are included. 8–10 NMJs were measured for each genotype and age (for details on number of flies analyzed, see Figs 2 and 4). The two subscripted numbers after F functions indicate the degrees of freedom for the between-group and within-group variance. For H function, the first subscript indicates the degrees of freedom.

Consistent with previous findings on the *Drosophila* larval NMJ and central adult synapses [34], overexpression of PI3K lead to a robust expansion of the adult NMJ, which showed increased number of synaptic contacts, boutons and branches at both ages relative to controls with normal PI3K levels (Figs 3 and 4). The ratio of synapses per bouton also increased (Fig 4D), supporting independent mechanisms to regulate bouton and active zone formation. Interestingly, the number of synaptic contacts decreased from 15 to 20 dae (Fig 4A), therefore following the pattern of age-dependent variations observed in controls.

Simultaneous expression of PI3K and any of the three Aβ peptides also produced an increase in the number of active zones, boutons and branches when compared to Aβ expressing NMJs with normal PI3K levels, demonstrating that overactivation of the PI3K pathway can induce synaptic overgrowth even in the presence of Aβ peptides (compare Figs 2 and 4; Table 3). However, our data demonstrate that Aβ peptides can still exert their synaptotoxic effects on synapses with elevated PI3K levels, because synaptic parameters were significantly reduced in NMJs co-expressing PI3K and any of the three amyloid peptides when compared to NMJs expressing PI3K in the absence of Aβ (Fig 4).

In general, specific Aβ effects on active zones in NMJs overexpressing PI3K followed a similar tendency to that observed with normal PI3K levels, providing further support for the specific effects uncovered in this study (compare Figs 2 and 4). For example, Aβ42arc induced the largest synaptic reduction also in the presence of elevated PI3K (Aβ42arc: 37.3±9.2% at 15 dae, 39.2±6.1% at 20 dae; Aβ40: 31.8±5.0% at 15 dae, 19.0±4.6% at 20 dae; Aβ42: 22.9±7.3% at 15 dae, 17.8±9.4% at 20 dae). However, some differences were detected when assessing how the level of PI3K activity modifies amyloid outcome. First, *post hoc* analyses showed that amyloid peptides induced significantly larger synaptic reduction in the presence of elevated PI3K levels (p<0.0001 for all three comparisons D42;UAS-Aβ* vs. D42;UAS-Aβ*, *UAS-PI3K*). Second, as with normal PI3K levels, Aβ42arc exerted the strongest effect on branch and boutons in conditions of elevated PI3K (Fig 4B and 4C), but deleterious effects of Aβ40 and Aβ42 on these structures also became significant when PI3K signaling was augmented (p<0.0001 for all three comparisons D42;UAS-Aβ* vs. D42;UAS-Aβ*, *UAS-PI3K*).

Noticeably, Aβ40 also altered the age-dependent synaptic pattern when PI3K was overexpressed, so that the number of active zones remained constant between 15 and 20 dae (Fig 4A). This confirms our initial observations with normal PI3K levels, and provides strong support for a specific role of Aβ40 in opposing synapse addition during synaptic refinement.

Our data show that the synaptotoxic effects of each Aβ peptide persist in NMJs overexpressing PI3K. Aβ peptides have been reported to inhibit the PI3K pathway both *in vivo* and in cell culture [25,44,45]. Hence, the observed Aβ induced synaptic reduction in flies with elevated PI3K may be mediated by inhibition of the PI3K synaptogenic pathway. We therefore compared the activity of the PI3K pathway in 15 day-old flies co-expressing PI3K and each Aβ peptide. For this purpose, we quantified the degree of phosphorylation of Akt, the main effector kinase of the pathway, and the level of inhibitory phosphorylation of GSK3β, a target of the pathway especially relevant to AD. Over expression of PI3K alone induced a marked increase of the ratio p-S505Akt/Akt when compared with flies with normal PI3K levels (Fig 4E). All three peptides showed a tendency to reduce this ratio, albeit this reduction was statistically significant only for the Aβ42arc peptide (Fig 4E). A similar trend was observed for GSK3β inhibitory phosphorylation, but differences were not statistically significant for any of the genotypes (Fig 4F). These data suggest that in NMJs with elevated PI3K levels, amyloid peptides can inhibit the PI3K pathway, opposing its synaptogenic activity. To test if a similar mechanism might explain the synaptotoxic effect of Aβ in NMJs with normal PI3K levels, we quantified inhibitory phosphorylation of GSK3β in flies expressing each of the three Aβ species, and found a significant reduction only for NMJs expressing Aβ42arc (Fig 4F). Together, our data suggest that in *Drosophila*, amyloid peptides can also block PI3K pathway activation, and that this inhibition contributes to synaptic loss.

### PI3K over expression can partially rescue early neurodegeneration induced by Aβ42, but not by Aβ42Arc

Our previous results show that Aβ40 and Aβ42-derived peptides display qualitatively different synaptotoxic effects, which may be partially mediated by inhibition of the PI3K pathway. Data gathered from various studies in *Drosophila* suggest that Aβ-induced synaptic deficit, cell loss, locomotor decline, and reduced life span may be elicited by different mechanisms [19,22,23,46,47], which could have different requirements for PI3K signaling. To gain insights into this question, we analyzed how PI3K over expression influences Aβ effects on neurodegeneration and life span.

**Fig 5:**
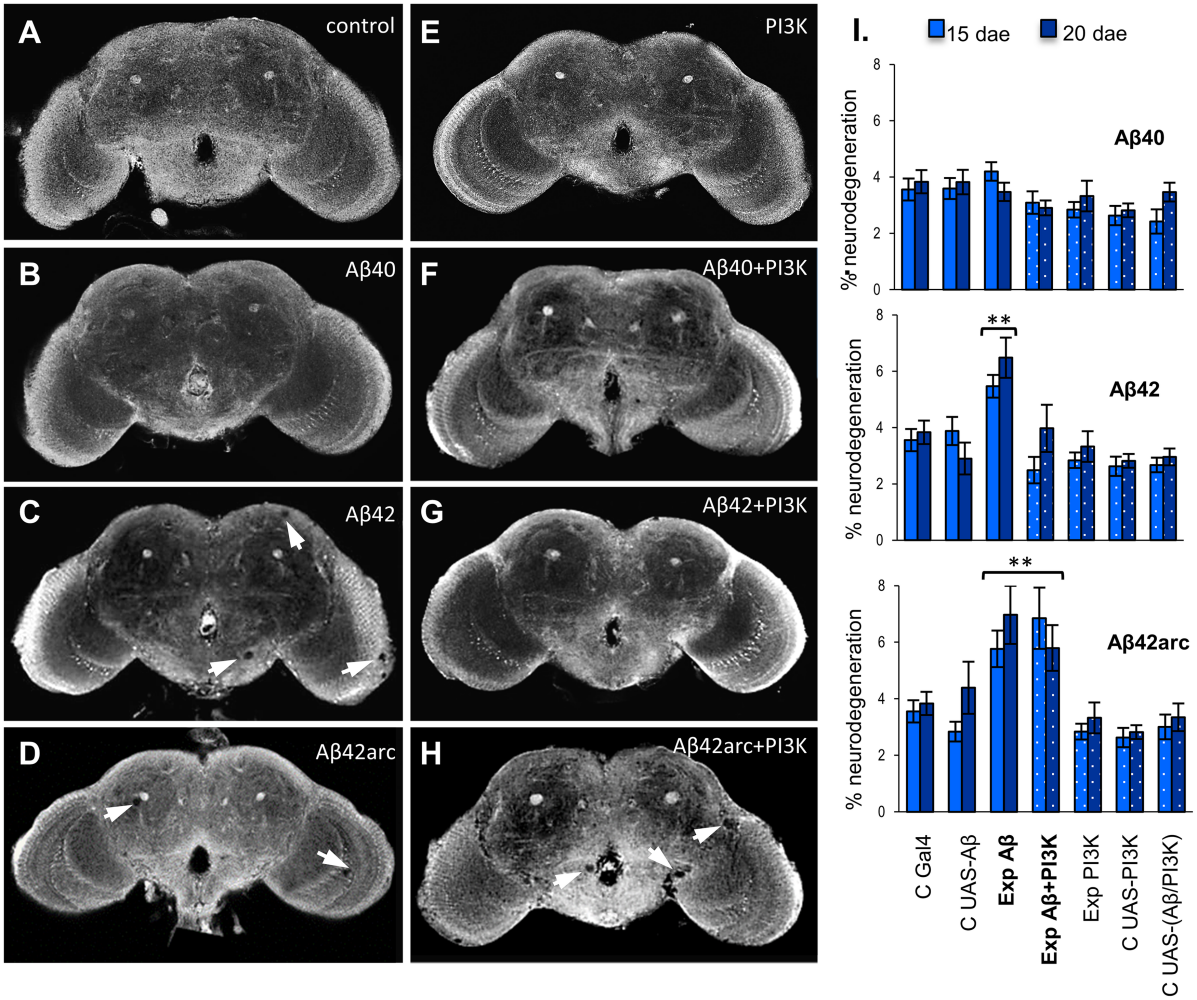
Effect of neuronal expression of three amyloid peptides, with or without elevated PI3K levels, on adult neurodegeneration. **(A-H)** Representative sections of cleared whole brains from 20-day old control males (A and E), and males expressing Aβ40 (B and F), Aβ42 (C and G), or Aβarc (D and H), with normal (A-D) or elevated (E-H) levels of PI3K. Arrows point to holes of neurodegenerated tissue. **(I)** The percentage of neurodegenerated area was measured at 15 and 20 days after eclosion (dae) in whole brains from males expressing Aβ40, Aβ42, or Aβ42arc with normal (Exp Aβ) or elevated levels of PI3K (Exp Aβ+PI3K), and control (C *) and PI3K-overexpressing (Exp PI3K) males. Control genotypes were as follows: C Gal4—*w;elav-Gal4/+*, C UAS-Aβ - w;UAS-Aβ*/+, C UAS-PI3K - *w;UAS-PI3K/+*, C UAS-(Aβ/PI3K)—w;UAS-PI3K UAS-Aβ*/+. Experimental genotypes were w; elav-Gal4 /UAS-Aβ* for Aβ-expressing flies, w; elav-Gal4 /UAS-PI3K UAS-Aβ* for flies co-expressing Aβ and high PI3K levels, and *w;elav-Gal4/UAS-PI3K* for flies with elevated PI3K. Each data point represents the average of 7–10 samples of the same genotype/age ±SEM. Logarithmic transformation of the data rendered a normal distribution, and thus was used for the ANOVA analysis, which showed a main effect of genotype (F_14,226_ = 10.044, p<0.001) and age (F_1,226_ = 4.156, p = 0.04), but not of their interaction (F_14,226_ = 1.370, p = 0.169). Asterisks denote significant differences (** p<0.01) with the other genotypes. Notice that over expression of PI3K can rescue neurodegeneration induced by Aβ42, but not by Aβ42arc.

Neuroprotective roles have been well established for PI3K [48]. Because possible neuroprotective effects would be easier to detect at early phases of the neurodegenerative process, we quantified neurodegeneration at relatively early ages (15 and 20 dae), using a pan-neural driver and an automated image analysis tool that measured brain tissue loss in whole brains. Indeed, this technique allowed detection of significant neurodegeneration as early as 15 dae with Aβ42-derived peptides (Fig 5), while prior experiments using very similar experimental conditions revealed first signs of neurodegeneration at 30 dae [22]. At these young ages, and despite the difference in peptide content (Fig 2F), the extension of the neurodegenerated area was similar in Aβ42- and Aβ42arc-expressing brains. Even though the two-way ANOVA detected significant age dependent differences, *post hoc* analyses did not reveal differences between 15 and 20 dae within each genotype. Flies expressing Aβ40 or PI3K, or both, were not significantly different from controls (Fig 5I), and thus did not evidence brain tissue loss.

Importantly, overexpression of PI3K was able to reduce Aβ42-induced neurodegeneration to almost wild type levels at both ages (Fig 5I). On the contrary, toxicity of Aβ42arc was not influenced by PI3K levels at any of the two ages tested (Fig 5I). These results are consistent with the level of PI3K pathway activity measured by Akt phosphorylation (Fig 4E), which show higher activity levels in Aβ42 than in Aβ42arc expressing flies, and suggest that activation of this pathway can protect against Aβ-induced cytotoxicity.

**Fig 6:**
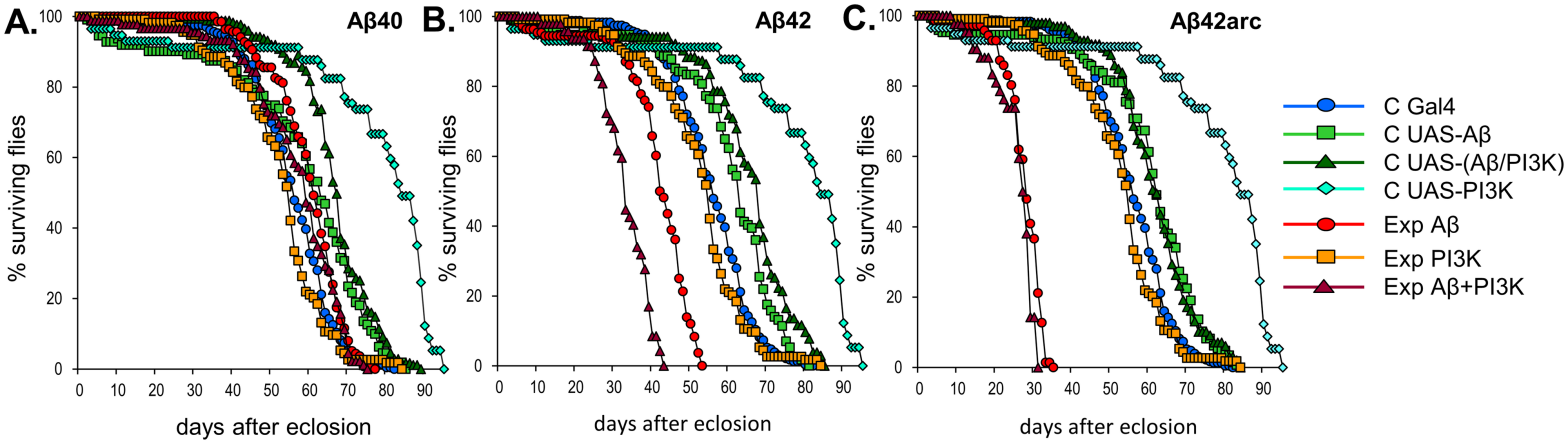
Effect of neuronal expression of three amyloid peptides, with or without overactivated PI3K signaling, on *Drosophila* life span. Life span was measured for males expressing Aβ40 **(A)**, Aβ42 **(B)**, or Aβ42arc **(C)** with normal (Exp Aβ: genotype w; D42-Gal4 /UAS-Aβ*) or elevated levels of PI3K (Exp Aβ+PI3K: genotype w; D42-Gal4 /UAS-PI3K UAS-Aβ*), and in control males (C Gal4: *w;D42-Gal4/+*, C UAS-Aβ: w;UAS-Aβ*/+, C UAS-PI3K: *w;UAS-PI3K/+*, C UAS-(Aβ/PI3K): w;UAS-PI3K UAS-Aβ*/+) and PI3K-overexpressing males (Exp PI3K: *w;D42-Gal4/UAS-PI3K*). Differences in survival were analyzed using Kaplan-Meier survival plots and log-rank analysis (see Results for details). For each genotype, at least five experiments were performed with an average of 20 flies/experiment.

We next tested if PI3K positively affected life span in males expressing each of the Aβ peptides. Although most controls showed undistinguishable cumulative survival curves (Fig 6), there were significant differences in some control lines which complicated interpretation of the results. *UAS-PI3K* control flies displayed an abnormally high survival (p<0.001 *vs*. all other genotypes), while *D42-Gal4* control males had slightly reduced survival when compared to the other control genotypes (p<0.001 *vs*. all other control genotypes). Life span of flies overexpressing PI3K alone was undistinguishable from the Gal4 control (χ^2^ = 1.03, p = 1), but lower than the other control genotypes (p<0.001).

Consistent with previous reports [21,22,26,46], expression of Aβ40 did not reduce life span, not even in the presence of overexpressed PI3K (Experiment Aβ40 *vs*. Experiment Aβ40+PI3K: χ^2^ = 0.22, p = 1)(Fig 6A). Furthermore, longevity of Aβ40 flies, with or without elevated PI3K levels, was higher than Gal4 controls (Control Gal4 *vs*. Experiment Aβ40: χ^2^ = 11.36, p = 0.011; Control Gal4 *vs*. Experiment Aβ40+PI3K: χ^2^ = 10.38, p = 0.018) and flies expressing PI3K alone (Experiment PI3K *vs*. Experiment Aβ40: χ^2^ = 16.65, p<0.001; Experiment PI3K *vs*. Experiment Aβ40+PI3K: χ^2^ = 14.95, p = 0.002), which points to a possible neuroprotective effect of this peptide. In contrast, Aβ42 significantly reduced survival rate (p<0.001 *vs*. all other genotypes)(Fig 6B), as has been previously shown [21–23,26,46]. Simultaneous expression of Aβ42 and PI3K reduced longevity even further (p<0.001 *vs*. all other genotypes)(Fig 6B), indicating a synergistic deleterious effect between the two. In line with earlier studies [22,23,25,26,47], Aβ42arc showed the highest toxicity in this assay, which was not affected by the level of PI3K expression (Experiment Aβ42arc *vs*. Experiment Aβ42arc+PI3K χ^2^ = 6.25, p = 0.1738; p<0.001 for both *vs*. all other genotypes)(Fig 6C). Thus, overexpression of PI3K does not seem to reduce Aβ toxicity in longevity assays. In summary, our data reveal that the implication of the PI3K pathway in the toxicity of Aβ differs for different Aβ species and in different contexts (synapse formation, cytotoxicity, and life span).

## Discussion

Synapse dysfunction and loss are key to dementia in Alzheimer’s disease (AD). It is currently established that amyloid-β peptides are important contributors to these synaptic alterations, but the precise identity of the Aβ species involved is not well defined [10,49]. Likewise, it is unclear how aging, the most influential non-genetic risk factor in AD, influences Aβ synaptotoxicity. These are relevant questions to designing suitable therapeutic strategies. We postulated that the *Drosophila* adult glutamaergic neuromuscular junction (NMJ) would provide an ideal system in which to address these issues, because its stereotypic morphology would allow uncovering subtle albeit relevant changes. Moreover, our work has revealed a temporal biphasic process of synaptic remodeling at the adult NMJ. An early phase of net synapse addition takes place during the first two weeks of adult life, a critical time period in which several areas of the young fly brain have been shown to undergo experience-dependent structural plasticity [29,50,51]. Thenceforth, net synaptic elimination occurs, consistent with the onset of behavioural and synaptic senescence [31,32,50,52]. Thus, this model allows assessing Aβ influence on synaptic dynamics during synaptic maturation and aging. In this model we show that: (1) Aβ40 seems to reduce the number of synaptic contacts by preventing synapse addition; (2) Aβ42 synaptotoxicity gradually increases with age; and (3) Aβ42arc produces net synaptic loss from early adulthood, but does not impede synapse addition. In summary, we demonstrate specific age-dependent synaptic effects for each Aβ peptide, and establish the fly adult NMJ as a suitable model to investigate the mechanisms underlying these peculiarities.

### Early amyloid synaptotoxicity or a physiological role for Aβ?

Studies in *Drosophila* had shown early memory defects induced by expression of both Aβ40 and Aβ42-derived peptides [19,20,22,23], but a structural and/or functional synaptic correlate had only been found for Aβ42-derived peptides [19,20,25,26]. Here, we show for the first time that Aβ40 reduces the number of synaptic contacts, and it seems to do so by preventing the addition of new synapses that normally occurs in the adult NMJ. At young ages (3–15 days), Aβ42 effect was remarkably similar to Aβ40, which suggests that early in life, Aβ42 may act similarly to Aβ40 by opposing synapse addition. However, Aβ42-expressing brains had the lowest amount of total peptide, which might explain these relatively mild early effects. In contrast, the mutant amyloid peptide Aβ42arc elicited net synaptic loss also at young ages, yet it showed a pattern of age-dependent variation in synapse number similar to controls, indicating that it does not hinder synapse addition. These are significant qualitative differences that point to a physiological role for wild type amyloid species in the process of synaptic plasticity.

In general, exposure to Aβ is associated with weakening of synapses, which is consistent with a postulated Aβ function in promoting activity-dependent synaptic elimination during mammalian postnatal development [16]. Our data suggest that rather than eliciting synapse removal, Aβ40, and possibly Aβ42, prevents the formation and/or maturation of new synapses in *Drosophila*. Interestingly, early defects in ocular dominance plasticity in *APPswe* transgenic mice, which generate elevated levels of wild type amyloid species, are associated with reduced strengthening and expansion of non-deprived eye cortical representations, while deprived eye weakening remains intact [53]. These data argue in favor of a similar effect for Aβ at synapses from juvenile mice and flies. In the *Drosophila* larval NMJ, the transition from immature to mature synapse involves changes in the relative contribution of specific glutamate receptors in postsynaptic receptor fields opposed to presynaptic active zones [54]. In mammals, synaptic elimination and maturation also involves changes in expression and trafficking of AMPA and NMDA glutamate receptors [55], a process that can be altered by Aβ [5,56–58]. The model described in this work represents an invaluable tool for genetically assessing the contribution of Glutamate receptors, and other molecules, in the amyloid-dependent alterations of synaptic refinement.

Although we cannot provide a mechanistic basis for the specific synaptotoxic activities of each Aβ species, the data on NMJs overexpressing PI3K suggest the direct implication of this pathway. First, despite its strong synaptogenic activity, PI3K over expression was not able to block Aβ-induced synaptic reduction. Second, western quantification showed that the activity of the pathway is reduced by Aβ expression, which is consistent with data from mammalian systems that suggest that amyloid peptides block Akt activation downstream of the PI3K enzyme [44,45], and increase GSK3 activity [43]. Moreover, the degree of PI3K pathway inhibition correlated with the extent of synaptic reduction, both being maximum for Aβ42arc. Altogether, evidences point to a direct relationship between Aβ and the PI3K/Akt/GSK3 pathway as central to Aβ-induced synaptic dysfunction, but further studies would be necessary to unambiguously prove it. The importance of this matter is underscored by the relevance of GSK3 in AD, which has been used as a therapeutic target [42,43].

### Aging and amyloid synaptotoxicity

The risk of AD increases with age, but the link between aging and Aβ toxicity is not fully understood. We have shown that after a stage of synaptic growth, synaptic elimination commences in the fly NMJ sometime between 15 and 20 days after eclosion. This age also represents a point of transition from growth to retraction for the abdominal longitudinal NMJ [27] and the mushroom bodies [30], and thus defines an age-dependent change in synaptic dynamics. Our data suggest that this age also delimits a change in the synaptic action of Aβ42, which would oppose synapse addition in early adulthood, but cause synaptic removal in aged flies. In contrast, Aβ42arc induces net synaptic loss at a similar rate at all ages tested, while Aβ40 maintains an unvarying number of active sites at least until 30 days. These data suggest that the impact of age on Aβ synaptotoxicity differs for each amyloid species. Understanding the mechanisms underlying age-dependent synaptic changes in wild type adult NMJs will be necessary for explaining the observed effects.

Several *in vitro* and *in vivo* studies have demonstrated different aggregation kinetics for the three Aβ species, which result in higher effective concentrations of specific aggregation forms with diverse toxic activities. Specifically, a recent *in vivo* study expressing tandem dimeric Aβ peptides in *Drosophila* has shown that the aggregation kinetics of Aβ42 favors a relatively high population of toxic oligomeric species, while this population is undetectable for Aβ40, which seems to more rapidly transit from monomeric to insoluble inert forms [59]. The E22G mutation in Aβ42arc has been shown to accelerate both Aβ oligomerization and fibrillogenesis [60,61]. Thus, it is tempting to speculate that Aβ monomeric forms inhibit synapse addition, while oligomeric forms promote synapse removal. Assessing the synaptic effects of the various tandem dimeric Aβ peptides [59] on the *Drosophila* adult NMJ would provide invaluable data to test this hypothesis.

### Amyloid-dependent synaptotoxicity, cytotoxicity, and life span shortening

Multiple studies in *Drosophila*, and in other models, suggest that the mechanisms underlying Aβ synaptotoxicity, cytotoxicity, and reduction of life span are different [19,22,23,46,47]. Even more, *in vivo* data demonstrate that manipulations that alter aggregation propensity can induce qualitative, rather than quantitative, shifts in the pathology induced [24]. Our data provide further support to this notion. First, we show that Aβ40 disturbs synapses, but not cell survival or life span. Second, neurodegeneration levels were similar in 20 day-old flies expressing either Aβ42 or Aβ42arc, yet both genotypes displayed dramatically different life expectancy. Third, over expression of PI3K reduced early Aβ42 cytotoxicity, measured by degree of neurodegeneration, but it enhanced the deleterious effects of this peptide on life span.

The differential effect of PI3K hyperactivation on Aβ-related phenotypic outcomes is intriguing. PI3K is an essential signaling pathway with multiple developmental and physiological functions; these include widespread functions such as regulation of cell proliferation and metabolism, or control of cellular remodeling and migration, but also cell-type specific roles such as synapse plasticity in neurons [62]. Moreover, different levels of pathway activation seems to trigger distinct responses [63,64]. In view of this complexity, we can only speculate on how PI3K alters Aβ-induced phenotypes. Our data show that PI3K overexpression had no influence on Aβ42arc-related phenotypes, a finding that might well be explained by the significant reduction in the activity of the PI3K pathway displayed by Aβ42arc-expressing flies. In contrast, the observed reduced neurodegeneration in flies co-expressing Aβ42 and PI3K could reflect residual hyper-activation of the pro-survival PI3K pathway, as suggested by western quantification. This hypothesis is consistent with studies in mice which suggest that low levels of Akt activity are sufficient to support neuronal survival responses [64]. However, PI3K over-expression had a negative effect on life span in flies expressing Aβ42. Lifespan is an extremely complex trait which may be particularly sensitive to unbalanced conditions, and age-dependent accumulation of toxic amyloid aggregates might further disturb PI3K and related signaling networks, advancing deterioration. These data underscore the complexity of Aβ toxicity and the necessity to test it at different levels when assessing the consequences of therapeutic approaches.

In summary, using an *in vivo* model we demonstrate that (1) different amyloid species disturb synapses by differentially influencing synapse addition or synapse elimination, (2) age-dependent changes in their synaptotoxicity are species-specific, and (3) the toxic actions of each Aβ peptide differ in different contexts. Furthermore, our work demonstrates the value of the *Drosophila* adult NMJ as an ideal *in vivo* model for understanding specific effects of amyloid peptides on synaptic plasticity.
